# Quality of life in children three and nine months after discharge from a paediatric intensive care unit: a prospective cohort study

**DOI:** 10.1186/1477-7525-6-21

**Published:** 2008-03-11

**Authors:** Hendrika Knoester, Madelon B Bronner, Albert P Bos, Martha A Grootenhuis

**Affiliations:** 1 Paediatric Intensive Care Unit, Emma Children's Hospital, Academic Medical Centre, Meibergdreef 9, 1105 AZ Amsterdam, The Netherlands; 2 Psychosocial Department, Emma Children's Hospital, Academic Medical Centre, Meibergdreef 9, 1105 AZ Amsterdam, The Netherlands

## Abstract

**Background:**

Improved survival in children with critical illnesses has led to new disease patterns. As a consequence evaluation of the well being of survivors of Pediatric Intensive Care Units (PICU) has become important. Outcome assessment should therefore consist of evaluation of morbidity, functional health and Health Related Quality of Life (HRQoL). Awareness of HRQoL consequences and physical sequelae could lead to changes in support during the acute phase and thereafter. The aim of this study was to evaluate HRQoL in PICU survivors.

**Methods:**

Prospective follow-up study three and nine months after discharge from a 14-bed tertiary PICU. Eighty-one of 142 eligible, previously healthy children were included from December 2002 through October 2005. HRQoL was assessed with the TNO-AZL Preschool Children Quality of Life Questionnaire (TAPQOL-PF) for children aged 1 to 6 years of age, the TNO-AZL Children's Quality of Life Questionnaire Parent Form (TACQOL-PF) for children aged 6 to 12 years of age, and the TNO-AZL Children's Quality of Life Questionnaire Child Form (TACQOL-CF) for children aged 8 to 15 years of age. The studied patients were compared with age appropriate normative data using non-parametric tests and effect sizes.

**Results:**

Thirty-one and 27 children, and 55 and 50 parents completed questionnaires respectively three and nine months after discharge. In 1–6 year old children parents reported more lung problems (3 and 9 months), worse liveliness (9 months) and better appetite and problem behaviour (3 months); in 6–12 year old children parents reported worse motor functioning (3 months); and 12–15 year old adolescents reported worse motor functioning (3 months). Large effect sizes indicating clinical significant differences in HRQoL with healthy control subjects were found on more domains.

**Conclusion:**

In this small group of PICU survivors differences in HRQoL with the normative population exist three and nine months after discharge. Calculated effect sizes were smaller nine months after discharge. These changes suggest that HRQoL improves over time. More research is necessary but we believe that HRQoL assessment should be incorporated in follow-up programs of PICU survivors.

## Background

The development of paediatric intensive care units (PICU's) has contributed to improved survival in children with critical illnesses. [1,2] As a consequence of improved survival new disease patterns evolved, such as growth disturbances of limbs after meningococcal disease. [3,4] Outcome assessment should therefore consist of evaluation of morbidity and well being. Functional health and Health Related Quality of Life (HRQoL) are used as outcome measures to evaluate patient's well being. In 1948, the World Health Organization defined health as 'a state of complete physical, mental and social well-being, and not merely the absence of disease or infirmity'. *Functional health *is defined as an individual's ability to perform normal daily activities, essential in order to meet basic needs, to fulfill usual roles, and to maintain health and well-being. [5]*Quality of life *(QoL) is defined as an individual's perception of their position in life, in the context of the culture and value systems in which they live, and in relation to their goals, expectations, standards and concerns. [5] HRQoL is defined as QoL in which a dimension of personal judgement over one's health and disease is added. [6] In case of children, HRQoL is influenced also by factors such as the ability to participate in peer groups and the ability to keep up with developmental activities. Several difficulties lie in measuring QoL in children, including (1) lack of consensus on suitable instruments, (2) the need for different instruments in different age groups and (3) the need of proxy reporting by parents or clinicians in children younger than 8 years of age. [7-11]

Although physical and psychological sequelae have been described, studies focusing on HRQoL in PICU survivors are scarce. [12-17] The Health Utilities Index (HUI) 2 is used in five studies to evaluate functional health. In three studies ± 30% of PICU survivors were in full health, one year after discharge. [18-20] In the Australian and Swiss studies using the HUI 2, ± 80% of the survivors were scored as having good quality of life one year after PICU discharge. [21,22] The HUI 2 evaluates only six domains of HRQoL: sensation, mobility, emotion, cognition, self-care and pain. Evaluation is done by the parents and not the children themselves when older than eight years of age. In another Australian study the Royal Alexandra Hospital for Children (RAHC) Measure of Function-Clinical Rating Scale was used to evaluate QoL: 60% of survivors had normal QoL, 3 to 24 months after discharge. [23] Evaluation is done by the primary physician. In all studies loss of follow-up is substantial and selection bias is possible. No studies exist, evaluating changes over time in HRQoL.

The aim of our study was to evaluate HRQoL in previously healthy children that were unexpectedly admitted to the PICU at two time points (three and nine months) after discharge and to evaluate whether HRQoL changes over time. Evaluation was done at the out-patient follow-up clinic by validated questionnaires completed by parents of children younger than 8 years of age and by children themselves when 8 years of age or older. We hypothesized that HRQoL is decreased after PICU discharge and improves over time.

## Methods

This study was part of an on-going explorative research program on physical and psychological sequelae and consequences for QoL in children and their parents after an acute and unexpected PICU admission. The PICU of the Emma Children's Hospital/Academic Medical Center Amsterdam is a tertiary PICU with 14 beds admitting patients from the greater Amsterdam area. Medical, surgical and trauma patients and patients from all pediatric subspecialties are admitted.

In this study we only wanted to include previously healthy children that were unexpectedly admitted to the PICU with a serious illness, for instance respiratory and circulatory insufficiency due to respiratory syncitial virus (RSV) infection or meningococcal disease, and all trauma patients. Furthermore children admitted for respiratory insufficiency necessitating ventilatory support for at least 24 hours and children admitted to the PICU for at least 7 days were included. We excluded children with known underlying illnesses or patients with scheduled elective surgery; admission due to abuse or self-intoxication and the inability to complete Dutch questionnaires because of a language barrier. The study was done from December 2002 through October 2005.

After discharge from the PICU, each family received a letter explaining the aim and content of the research program. Families were contacted by telephone to enhance participation. For cases lacking telephone contact, follow-up letters with tear-off reply slips inviting participation were sent. Written informed consent was obtained from all participating families. The Medical Ethic Committee of the Academic Medical Centre in Amsterdam has approved the study protocol.

Three and nine months after discharge QoL questionnaires were sent to all families with a reply envelope. All families were invited to visit the outpatient follow-up clinic three months after discharge for structured medical and psychological examination of the child by a pediatric intensivist and a psychologist.

### Health related quality of life questionnaires

**Figure 1:**
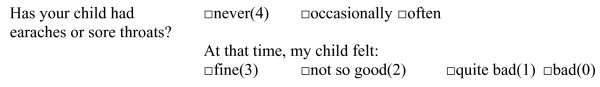
**Example of question in HRQoL questionnaire**. Numbers between brackets refer to the values resulting in the HRQoL scores.

HRQoL was assessed with the TNO-AZL Preschool Children Quality of Life Questionnaire (TAPQOL-PF) for children aged 1 to 6 years of age, the TNO-AZL Children's Quality of Life Questionnaire Parent Form (TACQOL-PF) for children aged 6 to 12 years of age, and the TNO-AZL Children's Quality of Life Questionnaire Child Form (TACQOL-CF) for children aged 8 to 15 years of age. [24-31] These questionnaires are generic Dutch instruments that measure health status problems weighted by the impact of the health status problems on well being. It offers the respondent the possibility of differentiating between their functioning and the way they feel about it. Most of the items consist of two questions linked to another. On the first one, the respondent can rate on a three point scale, (never, occasionally, or often), whether or not a specific problem occurred in the past few weeks. If a problem occurred, the child can indicate how it felt about this problem on a four point Likert scale: (very) good (3) – not so well (2) – rather bad (1) – bad (0). Numbers between brackets refer to the values resulting in the HRQoL scores. (Figure 1) The items are clustered into multi-item scales; scale scores are calculated by adding up item scores within scales, with higher scores indicating better QoL. For the TAPQOL-PF crude scales scores are transformed linearly to a 0–100 scale; for the TACQOL-PF and TACQOL-CF maximum domain scores are 16 for the emotional scales and 32 for the other scales. In the calculation of the scale scores one or two missing combined-item scores are allowed for. They are replaced by the mean value of the non-missing (combined) item scores. For respondents with more missing combined item scores per scale, the scale is assumed to be missing. Normative data from the general Dutch population are available. The instruments measure HRQoL on a group level in a reliable and valid way. Reliability (Cronbach's alpha) in the general populations are moderate to good (0.59–0.84) for all scales in all questionnaires, except for 'stomach functioning' and 'motor functioning' in the TAPQOL-PF. Criterion validity is good for all scales in all questionnaires, demonstrating that the scales can detect differences between healthy and less healthy children. [27,30,31] The studied patients were compared with age appropriate normative data using non-parametric tests and effects sizes.

The TAPQOL-PF assesses the child's functioning on 43 items in 12 domains: sleeping, appetite, lungs, stomach, skin, motor functioning, liveliness (together physical functioning), problem behavior, anxiety, positive mood (together emotional functioning), communication (cognitive functioning), and social functioning. Scales that measure motor functioning, social functioning and communication are applicable only to children 1.5 years and older.

The TACQOL-PF and TACQOL-CF assess functioning on seven domains: physical complaints, motor functioning, autonomy, cognitive functioning, social functioning (in relation to peers), positive and negative emotional functioning. Scale structure and reliability proved less satisfactory for the children between 12–15 years of age. Therefore some items from the original social scale were removed and the autonomy scale was removed in its totality. [31]

Patient characteristics were obtained from medical records and the Patient Data Management System.

### Statistics

Data analysis was done with the Statistical Package for Social Sciences (SPSS), Windows version 11.5. Before conducting the final analyses several preparation analyses were conducted: (1) Scales were constructed and missing data imputed on the basis of the guidelines of the questionnaires used; (2) descriptive statistics (Mann-Whitney and Chi-square tests) were used to describe the patient characteristics of the participants and non-participants; (3) non-parametric tests, (one sample sign-test or binomial test if median was equal to maximum), were performed to test whether the median or the binomial distribution of the several HRQoL-scales scores of the patients differed from the normative data available; (4) effect sizes (d) were calculated by dividing the difference in mean scores between the patients and the normative group by the standard deviation of the scores in the normative group. According to Cohen, effect sizes of about 0.2 were considered to be small, effect sizes of about 0.5 to be moderate, and effect sizes of about 0.8 to be large [32]; (5) all above mentioned analyses were performed for the whole group of participants (completed questionnaires 3 and/or 9 months after discharge); (6) paired t-tests were performed for the group that completed both questionnaires, to test for differences over time. A significance level of p < 0.005 was used in all tests to compensate for multiple testing.

Patients' self-reports were used for analysis unless the self-report was not available due to the young age of the patient. Separate analyses were conducted for (1) patients aged 1–5 years, using the TAPQOL-PF, (2) patients aged 6–11 years, using the TACQOL-PF, (3) patients aged 8–11 years, using the TACQOL-CF for children, and (4) patients aged 12–15 years, using the TACQOL-CF for adolescents. We decided to report all age groups despite small numbers, because the results in the different age groups were comparable.

## Results

### Participants

**Figure 2:**
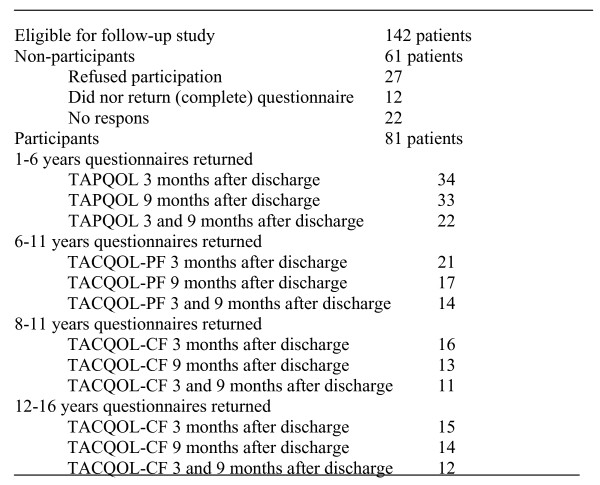
**Participants and non-participants**. Numbers of participants that completed the different questionnaires at 3 and 9 months after discharge and numbers of non-participants.

A total of 142 patients were eligible for participation in this part of the study. Twenty-seven refused participation (for geographical reasons or no interest), 12 said that they would like to participate but never returned their questionnaires or did not complete the full questionnaire; 22 families did not respond at all. Of 81 children either the children or their parents completed one or both questionnaires. The response rate is 57%. Thirty-one and 27 children completed questionnaires respectively three and nine months after discharge; 55 and 50 parents completed questionnaires respectively three and nine months after discharge. Twenty-three children and 36 parents completed questionnaires at both moments. (Figure 2) Statistically significant differences were found between participants and non-participants with respect to length of stay in the PICU. Fourteen participants and no non-participants had lengths of stay in the PICU of longer than 21 days. (Table 1).

**Table 1:** Patient characteristics of participants and non-participants

	Participants n = 81	Non-participants n = 61
	Median (range)	Median (range)

Age of child at PICU admission(yrs)	5.8 (1–14.9)	4.9 (1–14.8)
Length of stay in PICU (days)	5.0 (1–305)*	2.0 (1–31)
Length of artificial ventilation (days)	4.0 (0–43)	2.0 (0–26)
Risk of Mortality, PIM 2 (%)	4.4 (0–32.4)	3.8 (0–80.5)

	n (%)	n (%)

Male	44 (54)	36 (59)
Reason for PICU admission		
Respiratory insufficiency	34 (42)	31 (51)
Circulatory insufficiency	18 (22)	12 (20)
Trauma	29 (36)	18 (29)
Treatment characteristics (yes)		
Artificial ventilation	62 (76)	45 (74)
Circulatory support	22(27)	12 (20)

* p < 0.05 participants versus non-participants

### TAPQOL-PF 1–6 years of age

Thirty-four and 33 of 75 eligible parents completed the TAPQOL-PF respectively three and nine months after discharge, 22 of these parents completed the TAPQOL-PF at both moments. The response rate in this group is 45% at three months and 44% at nine months. Statistically significant differences between the PICU survivors and healthy control subjects were found on three domains (more lung problems, better appetite and better problem behaviour) three months after discharge and on two domains (more lung problems, worse liveliness) nine months after discharge. Large effect sizes were found on three domains nine months after discharge: indicating worse HRQoL on motor functioning, positive mood and liveliness. (Table 2)

**Table 2:** TAPQOL-PF. Mean HRQoL scores for children aged 1 to 6 years of age that completed one or both questionnaires (mean ± sd)

	3 months after discharge (n = 34)	Effect size (d)	P Value	9 months after discharge (n = 33)	Effect size (d)	P Value	Normative data
Stomach problems	91.9 ± 12.0	0.00	0.2	89.1 ± 15.8	0.3	0.2	91.9 ± 13.8
Skin problems	93.9 ± 8.9	0.2	0.3	91.9 ± 11.1	0.009	0.7	91.8 ± 10.8
Lung problems	83.8 ± 18.4*	0.6	0.000	85.9 ± 21.7*	0.5	0.002	93.6 ± 16.2
Sleeping	73.9 ± 21.1	0.5	0.03	78.5 ± 20.3	0.2	0.4	82.3 ± 17.3
Appetite	89.8 ± 10.0*	0.4	0.004	84.1 ± 15.5	0.04	0.08	84.6 ± 13.2
Motor functioning	95.7 ± 8.0	0.6	0.02	92.4 ± 15.4	1.4	0.007	98.5 ± 4.4
Social functioning	93.2 ± 15.1	0.1	0.2	88.7 ± 17.0	0.2	0.3	91.3 ± 15.4
Problem behaviour	76.6 ± 16.0*	0.6	0.004	73.4 ± 18.2	0.4	0.6	67.7 ± 15.3
Communication	90.8 ± 12.0	0.1	1.0	90.8 ± 13.4	0.1	0.6	91.7 ± 9.9
Anxiety	72.2 ± 21.5	0.3	0.5	79.6 ± 21.4	0.1	0.3	78.3 ± 18.0
Positive mood	97.4 ± 9.6	0.2	0.2	93.4 ± 16.6	0.8	0.01	98.7 ± 6.5
Liveliness	92.9 ± 16.2	0.6	0.03	90.6 ± 17.9*	0.9	0.002	98 ± 8.0

* p < 0.005 whole group versus normative data.

High scores represent a better HRQoL

### TACQOL-PF 6–12 years of age

Twenty-one and 17 of 38 eligible parents completed the TACQOL-PF respectively three and nine months after discharge; 14 of these parents completed the TACQOL-PF at both moments. The response rate in this group is 55% at three months and 45% at nine months. Statistically significant differences between PICU survivors and healthy control subjects were found on one domain (motor functioning) three months after discharge and on no domains nine months after discharge. Large effect sizes were found on four domains (indicating worse HRQoL on physical functioning, motor functioning, autonomy, and social functioning) three months after discharge and on one domain (indicating worse HRQoL on motor functioning) nine months after discharge. (Table 3)

**Table 3:** TACQOL-PF. Mean HRQoL scores for children aged 6–12 years of age that completed one or both questionnaires (mean ± sd)

	3 months after discharge (n = 21)	Effect size (d)	P Value	9 months after discharge (n = 17)	Effect size (d)	P Value	Normative data
Physical functioning	24.0 ± 4.7	0.8	0.02	25.9 ± 3.7	0.3	0.2	27.1 ± 4.0
Motor functioning	27.4 ± 4.6*	1.3	0.003	28.6 ± 5.4	0.8	0.05	30.8 ± 2.6
Autonomy	29.0 ± 4.2	1.3	0.01	31.1 ± 1.7	0.006	0.6	31.2 ± 1.7
Cognitive functioning	27.8 ± 4.8	0.3	0.8	27.4 ± 7.6	0.4	0.8	29.0 ± 3.8
Social functioning	27.9 ± 3.9	0.8	0.3	29.6 ± 3.4	0.1	0.4	29.9 ± 2.5
Positive emotions	13.9 ± 2.8	0.5	0.2	13.4 ± 3.5	0.7	0.5	14.8 ± 2.0
Negative emotions	10.1 ± 2.3	0.6	0.01	10.8 ± 3.3	0.3	0.6	11.5 ± 2.4

* p < 0.005 whole group versus normative data.

High scores represent a better HRQoL

### TACQOL-CF 8–12 years of age

Sixteen and 13 of 29 eligible children completed the TACQOL-CF respectively three and nine months after discharge; 11 of these children completed the TACQOL-CF at both moments. The response rate in this group is 55% at three months and 45% at nine months. No statistically significant differences between PICU survivors and healthy control subjects were found three and nine months after discharge. No large effect sizes were found three and nine months after discharge. (Table 4)

**Table 4:** TACQOL-CF 8–12. Mean HRQoL scores for children aged 8–12 years of age that completed one or both questionnaires (mean ± sd)

	3 months after discharge (n = 16)	Effect size (d)	P Value	9 months after discharge (n = 13)	Effect size (d)	P Value	Normative data
Physical functioning	22.4 ± 6.0	0.5	0.2	25.2 ± 5.0	0.03	0.5	24.9 ± 5.1
Motor functioning	28.6 ± 3.1	0.3	0.1	29.8 ± 3.3	0.009	0.2	29.8 ± 3.2
Autonomy	30.9 ± 2.4	0.2	0.5	31.3 ± 1.6	0.05	0.5	31.2 ± 1.9
Cognitive functioning	27.9 ± 4.6	0.2	1.0	30.8 ± 1.9	0.6	1.0	28.4 ± 3.9
Social functioning	30.9 ± 1.9	0.4	1.0	29.8 ± 3.4	0.02	0.8	29.7 ± 2.8
Positive emotions	12.1 ± 3.2	0.6	0.5	12.7 ± 3.1	0.4	0.7	13.6 ± 2.5
Negative emotions	9.8 ± 2.9	0.7	0.007	12.8 ± 2.6	0.4	0.1	11.6 ± 2.7

* p < 0.005 whole group versus normative data.

High scores represent a better HRQoL

### TACQOL-CF 12–16 years of age

Fifteen and 14 of 38 eligible adolescents completed the TACQOL-CF respectively three or nine months after discharge; 12 of these adolescents completed the TACQOL-CF at both moments. The response rate in this group is 40% at three months and 37% at nine months. Statistically significant differences between PICU survivors and healthy control subjects were found on one domain (motor functioning) three months after discharge and on no domains nine months after discharge. Large effect sizes were found on one domain three and nine months after discharge: indicating worse HRQoL on motor functioning. (Table 5)

**Table 5:** TACQOL-CF 12–15. Mean HRQoL scores for adolescents aged 12–15 years of age that completed one or both questionnaires (mean ± sd)

	3 months after discharge (n = 15)	Effect size (d)	P Value	9 months after discharge (n = 14)	Effect size (d)	P Value	Normative data
Physical functioning	22.9 ± 6.2	0.1	0.6	23.3 ± 6.1	0.07	1.0	23.7 ± 5.4
Motor functioning	24.7 ± 5.2*	1.6	0.001	26.8 ± 6.7	0.9	0.02	29.8 ± 3.2
Cognitive functioning	27.9 ± 3.8	0.1	1.0	28.5 ± 2.3	0.2	0.7	27.6 ± 4.1
Peers	30.5 ± 2.7	0.2	0,2	30.7 ± 2.6	0.1	0.2	31.1 ± 2.9
Positive emotions	11.8 ± 3.4	0.4	0.09	12.3 ± 2.4	0.2	0.1	13.0 ± 2.8
Negative emotions	10.4 ± 3.6	0.5	0.4	12.2 ± 3.5	0.2	0.8	11.6 ± 2.6

* p < 0.005 whole group versus normative data.

High scores represent a better HRQoL

### Differences over time

In the parents and children that completed both questionnaires, no statistically significant differences were found over time, indicating that HRQoL did not change from three to nine months after discharge.

## Discussion

This is one of the first studies to describe HRQoL of children after a PICU experience using validated and reliable instruments. In all age groups, except for 8–12 year old children statistically significant differences with the normative population were found on a number of domains. The parents of 1–6 year old children reported statistically significant lower scores on lung problems and liveliness; and statistically significant higher scores on emotional domains like problem behaviour and appetite. The parents of 6–12 year old children and the 12–15 year old children scored statistically significant lower on motor functioning. In the children and parents that completed questionnaires three and nine months after discharge no statistically significant differences were found over time. However, almost all differences with the healthy Dutch population disappeared after nine months, except for worse motor functioning in almost all age groups and more lung problems, worse positive mood and worse liveliness in 1–6 year old children.

Effect sizes (d) were calculated to assess the magnitude of differences in mean scores between the patient sample and the normative population. [32] In all evaluated groups moderate and large effect sizes were found on more HRQoL domains than statistically significant differences were found. At three months after discharge, effect size calculation indicated worse HRQoL on physical, social and emotional domains compared to the normative population. At nine months after discharge most effect sizes decreased, indicating improvement of HRQoL, except for motor functioning, positive mood and liveliness in 1–6 year old children. Probably statistically significant differences were not found due to the small number of evaluated patients in the different age groups. Despite the lack of statistically significant differences, changes in effect size suggest that several domains of HRQoL are decreased at three months after discharge and improve over time.

Studies focusing on QoL in PICU survivors are scarce. The Health Utilities Index (HUI) 2 was used in 4 studies. [18-21] In three of these studies 64% of the evaluated children reported no affected domains one year after discharge. [18,19,21] The HUI 2 is a questionnaire answered by the physician and not the child itself or its parents; and it does not measure all aspects of QoL. Mobility is the only physical aspect that is evaluated and social aspects, family functioning, and well being are not evaluated. In a fifth study the Royal Alexandra Hospital for Children (RAHC) measure of function was used. [23] In this study results of the telephone interviews of the physicians are reported. Consensus on appropriate questionnaires to evaluate HRQoL is essential. The ideal questionnaire should measure all aspects of QoL or HRQoL and if possible by the child himself (≥ 6–8 years of age). Proxy investigation (necessary if a child is <6–8 years of age) is second best because proxy reports do not always correspond with self-evaluations, depending on the health aspect being examined. For example, concordance for items and domains concerning functional limitations are higher compared to items and domains concerning emotional and social well being. [9,28,33] Physicians are not the right persons to answer QoL and HRQoL questionnaires of their patients. [8,34]

In the young children for whom parents completed the questionnaires, more statistically significant differences and larger effect sizes are found on HRQoL domains than in the older children that completed the questionnaires themselves. This study was not designed to compare self reporting and proxy reporting. In the 6–12 year old children that were evaluated by their parents moderate to large effect sizes were found in all domains except cognitive functioning, three months after discharge. In the 8–12 year old children that completed the questionnaires themselves moderate effect sizes were only found in physical functioning, and positive and negative emotional functioning, three months after discharge. A possible reason why parents report more problems, could be a different appraisal of the severity of the situation and consequently a different perception of HRQoL domains. It is imaginable that pediatric children look less into the future than their parents, and are less aware of the stressful situation that has occurred. Older children are possibly more aware what has happened, which leads to feelings of happiness to have survived. [9,28,33]

In the 1–6 year old children the domains appetite and problem behaviour are scored better than the normative population. An explanation of these results could be response shift. Patients and parents confronted with a life-threatening disease are faced with the necessity to accommodate to the illness. An important mediator of this process is response shift. Response shift means that the experience changes the internal standard of patients, resulting in changes in the meaning of self-evaluation and hence in a possibly different experience of problems and values, such as HRQoL. HRQoL studies are influenced by response shift. [35-37]

In the 1–6 year old children large and moderate effect sizes are found nine months after discharge in four domains: more lung problems, worse motor functioning, worse positive mood and worse liveliness. Apparently, parents of these young children report more physical and emotional problems than parents of healthy peers. HRQoL questionnaires or other measures on psychosocial functioning should be used to clarify problems as they are experienced by parents after discharge. We think that this finding needs further research.

It is important to notice that children and adolescents report more negative emotions compared to their healthy peers three months after discharge. Negative emotions included in the questionnaire are feelings of irritation or anger. Although the differences were not statistically significant we think that this finding needs further research. Surprisingly children and adolescents did not report a worse HRQoL for physical symptoms. (Table 4 and 5) Apparently children show physical recovery after PICU survival but the emotional impact may be substantial.

A number of limitations to this study should be taken into account. *First*, a considerable number of children were lost due to non-response and refusal. Other follow-up studies in the PICU have had similar response rates. [20] Due to the small number of children in the different age groups statistically significant differences with the normative population were scarce. *Second*, of the patients that participated an even smaller number fulfilled questionnaires at both moments. Possibly due to small numbers, we did not see effects over time. *Third*, we probably failed to see a number of patients whose parents were experiencing psychological problems, such as avoidance and refusal to come back to the hospital, a well known characteristic of Post Traumatic Stress Disorder. [12,14,38]*Fourth*, participants had a statistically significant longer stay in the PICU. Probably this is due to a small number of participants that were admitted longer than 21 days and therefore had more commitment to the PICU which might have influenced them to participate in the study. *Fifth*, we included a predominantly Caucasian Dutch population. The results of this study are not necessarily to be extrapolated to patients from other ethnic or cultural communities.*Sixth*, the results of this study are influenced by known limitations of the used questionnaires. The questionnaires have been described in manuals with appropriate normative populations. Cronbach's alpha's in all normative populations are moderate to good. For some scales reliability has been rather low, which may have been due to the low prevalence and variance of problems in the general population sample. The questionnaires are designed primarily for research purposes and focus mainly on data aggregated on the group level, for example in clinical trials, evaluative or descriptive studies. Thus, all scales can be used for group comparisons in a valid and reliable way. *Finally*, considering the small numbers of patients in the different age groups we have not been able to study the determinants of HRQoL. In future research it is important to pay attention to determinants of HRQoL of PICU survivors, to identify those most at risk for problems.

## Conclusion

In this small group of PICU survivors differences in HRQoL with the normative population exist three and nine months after discharge. Calculated effect sizes were smaller nine months after discharge. These changes suggest that HRQoL improves over time. Apparently PICU admission impacts HRQoL three and nine months after discharge. More research is necessary but we believe that HRQoL assessment should be incorporated in follow-up programs of PICU survivors. It should include (1) HRQoL evaluation, (2) evaluation of risk factors for decreased HRQoL and (3) support after discharge if needed. Because awareness of HRQoL consequences and physical sequelae could lead to changes in treatment during the acute phase, pediatric intensivists should be core members of PICU follow-up programs.

## Abbreviations

Child Form (CF), Health Related Quality of Life (HRQoL), Health Utilities Index 2 (HUI 2), Parent Form (PF), Pediatric Intensive Care Unit (PICU), Quality of Life (QoL), Royal Alexandra Hospital for Children (RAHC), respiratory syncitial virus (RSV), Statistical Package for Social Sciences (SPSS), TNO-AZL Children's Quality of Life Questionnaire (TACQOL), TNO-AZL Preschool Children Quality of Life (TAPQOL).

## Competing interests

The author(s) declare that they have no competing interests.

## Authors' contributions

HK drafted the manuscript, HK and MBB collected the data for this study, HK, MBB and APB contributed to the analysis and interpretation of data and wrote the manuscript, MAG designed the study, contributed to the interpretation of the data and critical revision of the manuscript. All authors read and approved the final version of the manuscript.

## References

[B1] Thorburn K, Baines P, Thomson A, Hart CA (2001). Mortality in severe meningococcal disease. Arch Dis Child.

[B2] Tilford JM, Roberson PK, Lensing S, Fiser DH (1998). Differences in pediatric ICU mortality risk over time. Crit Care Med.

[B3] Bache CE, Torode IP (2006). Orthopaedic sequelae of meningococcal septicemia. J Pediatr Orthop.

[B4] Belthur MV, Bradish CF, Gibbons PJ (2005). Late orthopaedic sequelae following meningococcal septicaemia. A multicentre study. J Bone Joint Surg Br.

[B5] Eiser C, Morse R (2001). Quality-of-life measures in chronic diseases of childhood. Health Technol Assess.

[B6] Eiser C (1997). Children's quality of life measures. Arch Dis Child.

[B7] Eiser C, Morse R (2001). The measurement of quality of life in children: past and future perspectives. J Dev Behav Pediatr.

[B8] Janse AJ, Uiterwaal CS, Gemke RJ, Kimpen JL, Sinnema G (2005). A difference in perception of quality of life in chronically ill children was found between parents and pediatricians. J Clin Epidemiol.

[B9] Jokovic A, Locker D, Guyatt G (2004). How well do parents know their children? Implications for proxy reporting of child health-related quality of life. Qual Life Res.

[B10] Rajmil L, Herdman M, Fernandez De Sanmamed MJ, Detmar S, Bruil J, Ravens-Sieberer U, Bullinger M, Simeoni MC, Auquier P (2004). Generic health-related quality of life instruments in children and adolescents: a qualitative analysis of content. J Adolesc Health.

[B11] Varni JW, Burwinkle TM, Lane MM (2005). Health-related quality of life measurement in pediatric clinical practice: an appraisal and precept for future research and application. Health Qual Life Outcomes.

[B12] Balluffi A, Kassam-Adams N, Kazak A, Tucker M, Dominguez T, Helfaer M (2004). Traumatic stress in parents of children admitted to the pediatric intensive care unit. Pediatr Crit Care Med.

[B13] Dahlem P, De Jongh FHC, Griffioen RW, Bos AP, Van Aalderen WMC (2005). Respiratory sequelae after acute hypoxemic respiratory failure in children with meningococcal septic shock. Crit Care Shock.

[B14] Ehrlich TR, Von R, Grootenhuis MA, Gerrits AI, Bos AP (2005). Long-term psychological distress in parents of child survivors of severe meningococcal disease. Pediatr Rehabil.

[B15] Madagame ET, Havens PL, Bresnahan JM, Babel KL, Splaingard ML (1995). Survival and functional outcome of children requiring mechanical ventilation during therapy for acute bacterial meningitis. Crit Care Med.

[B16] Rees G, Gledhill J, Garralda ME, Nadel S (2004). Psychiatric outcome following paediatric intensive care unit (PICU) admission: a cohort study. Intensive Care Med.

[B17] Robertson CM, Joffe AR, Moore AJ, Watt JM (2002). Neurodevelopmental outcome of young pediatric intensive care survivors of serious brain injury. Pediatr Crit Care Med.

[B18] Gemke RJ, Bonsel GJ, van Vught AJ (1995). Long-term survival and state of health after paediatric intensive care. Arch Dis Child.

[B19] Jayshree M, Singhi SC, Malhi P (2003). Follow up of survival and quality of life in children after intensive care. Indian Pediatr.

[B20] Jones S, Rantell K, Stevens K, Colwell B, Ratcliffe JR, Holland P, Rowan K, Parry GJ (2006). Outcome at 6 months after admission for pediatric intensive care: a report of a national study of pediatric intensive care units in the United kingdom. Pediatrics.

[B21] Taylor A, Butt W, Ciardulli M (2003). The functional outcome and quality of life of children after admission to an intensive care unit. Intensive Care Med.

[B22] Ambuehl J, Karrer A, Meer A, Riedel T, Schibler A (2007). Quality of life of survivors of paediatric intensive care. Swiss Med Wkly.

[B23] Morrison AL, Gillis J, O'Connell AJ, Schell DN, Dossetor DR, Mellis C (2002). Quality of life of survivors of pediatric intensive care. Pediatr Crit Care Med.

[B24] Bosch AM, Grootenhuis MA, Bakker HD, Heijmans HS, Wijburg FA, Last BF (2004). Living with classical galactosemia: health-related quality of life consequences. Pediatrics.

[B25] Bunge EM, Essink-Bot ML, Kobussen MP, van Suijlekom-Smit LW, Moll HA, Raat H (2005). Reliability and validity of health status measurement by the TAPQOL. Arch Dis Child.

[B26] Fekkes M, Theunissen NC, Brugman E, Veen S, Verrips EG, Koopman HM, Vogels T, Wit JM, Verloove-Vanhorick SP (2000). Development and psychometric evaluation of the TAPQOL: a health-related quality of life instrument for 1-5-year-old children. Qual Life Res.

[B27] Fekkes M, Bruil J, Vogels T (2003). TAPQOL-manual.

[B28] Theunissen NC, Vogels TG, Koopman HM, Verrips GH, Zwinderman KA, Verloove-Vanhorick SP, Wit JM (1998). The proxy problem: child report versus parent report in health-related quality of life research. Qual Life Res.

[B29] Verrips GH, Vogels TGC, Verloove-Vanhorick SP, Fekkes M, Koopman H, Kamphuis RP (1998). Health-related quality of life measure for children - The TACQOL. J Appl Ther.

[B30] Vogels T, Verrips GH, Koopman HM (2000). TACQOL manual Parent Form and Child Form.

[B31] Vogels T, Bruil J, Koopman H (2004). TACQOL CF 12-15 Manual.

[B32] Cohen J (1988). Statistical power analysis for the behavioral sciences.

[B33] Verrips GH, Stuifbergen MC, den Ouden AL, Bonsel GJ, Gemke RJ, Paneth N, Verloove-Vanhorick SP (2001). Measuring health status using the Health Utilities Index: agreement between raters and between modalities of administration. J Clin Epidemiol.

[B34] Janse AJ, Gemke RJ, Uiterwaal CS, van T, Kimpen JL, Sinnema G (2004). Quality of life: patients and doctors don't always agree: a meta-analysis. J Clin Epidemiol.

[B35] De CM, Regier D, Alamgir AH, Anis AH, Fitzgerald MJ, Marra CA (2005). Evaluating health-related quality-of-life studies in paediatric populations: some conceptual, methodological and developmental considerations and recent applications. Pharmacoeconomics.

[B36] Schwartz CE, Sprangers MA (1999). Methodological approaches for assessing response shift in longitudinal health-related quality-of-life research. Soc Sci Med.

[B37] Sprangers MA, Schwartz CE (1999). Integrating response shift into health-related quality of life research: a theoretical model. Soc Sci Med.

[B38] Board R, Ryan-Wenger N (2002). Long-term effects of pediatric intensive care unit hospitalization on families with young children. Heart Lung.

